# Berberine a traditional Chinese drug repurposing: Its actions in inflammation-associated ulcerative colitis and cancer therapy

**DOI:** 10.3389/fimmu.2022.1083788

**Published:** 2022-12-06

**Authors:** Cuipeng Zhu, Kaiqi Li, Xiao-Xu Peng, Tong-Jia Yao, Zi-Yu Wang, Ping Hu, Demin Cai, Hao-Yu Liu

**Affiliations:** ^1^ College of Animal Science and Technology, Yangzhou University, Yangzhou, China; ^2^ Joint International Research Laboratory of Agricultural & Agri-Product Safety, The Ministry of Education of China, Yangzhou University, Yangzhou, China

**Keywords:** berberine, ulcerative colitis, inflammation, microbes, cancer

## Abstract

Berberine (BBR), an isoquinoline alkaloid extracted from Coptidis Rhizoma, has a long history of treating dysentery in the clinic. Over the past two decades, the polytrophic, pharmacological, and biochemical properties of BBR have been intensively studied. The key functions of BBR, including anti-inflammation, antibacterial, antioxidant, anti-obesity, and even antitumor, have been discovered. However, the underlying mechanisms of BBR-mediated regulation still need to be explored. Given that BBR is also a natural nutrition supplement, the modulatory effects of BBR on nutritional immune responses have attracted more attention from investigators. In this mini-review, we summarized the latest achievements of BBR on inflammation, gut microbes, macrophage polarization, and immune responses associated with their possible tools in the pathogenesis and therapy of ulcerative colitis and cancer in recent 5 years. We also discuss the therapeutic efficacy and anti-inflammatory actions of BBR to benefit future clinical applications.

## Introduction

In modern medicine, natural products are closely linked to numerous health complications treatment and great therapeutic approaches. In particular, the functional metabolites derived from plants are suggested to perform various biological activities involving anti-obesity, anti-inflammation, antibacterial, anti-fatty liver, and anti-cancer (1, 2). Alkaloids act as the chemical defense in plants when producing secondary metabolites with beneficial pharmacological roles and account for 3/5 of plant-derived medicals. The bioactive components of alkaloids have been used for immunomodulatory therapeutic potentials and anti-inflammation (3). Among them, berberine (BBR) is an isoquinoline alkaloid purified from Chinese herbs and a naturally occurring compound extracted from *Coptidis Rhizoma* (4). In recent years, a series of actions of BBR-mediated anti-intestinal diseases, anti-cancer, antioxidative stress, and anti-inflammatory has been reported *in vivo* and *in vitro* (5–7). Indeed, intestinal disorders and cancer are tightly associated and always characterized by inflammation, oxidative stress, and a couple of immune outputs (8). Inflammation response of the body would benefit the recovery when exposed to infection or invasion events. In this process, immune cells are stimulated by BBR to fight the inflammatory responses in these diseases (9). It has been reported that the potential mechanisms of the anti-inflammation of alkaloids would be attributed to the inhibition of several pro-inflammatory enzyme complexes enrolled in inflammatory signaling processes (10). Moreover, evidence indicates that BBR could ameliorate intestinal lesions and tumor development by reducing macrophage and oxidative stress inflammatory responses (11). The possible underlying mechanisms, in particular, of signaling pathways, have been documented (12). It is plausible that at least part of the observed anti-inflammation roles is due to the activations of classic inflammatory signaling factors, including adenosine monophosphate-activated protein kinase (AMPK) and Wnt/β-catenin (13). Additionally, the extracellular signal-regulated protein kinase 1/2 (ERK1/2) (14), signal transducer and activator of transcription 1 (STAT1), protein kinase B (AKT), nuclear factor kappa-light-chain-enhancer of activated B cells (NF-kB), and nitric oxide (NO), prostaglandin E2 (PEG2), along with chemokines and cytokines (15–18) have also been illustrated. Again, the modulation of gut microbes and microphage polarization has previously been registered in BBR-mediated inflammatory regulation (19). This mini-review aims to collect updated information on BBR in the fields of inflammation and immune responses for a better understanding of the potential mechanisms in the pathogenesis process of various human and animal ulcerative colitis (UC) and cancer research. This will encourage researchers to explore further addressing all aspects of the utilization of BBR for new treatments and therapeutic strategies.

### BBR in ulcerative colitis-the anti-inflammatory, immunomodulatory effects and potential mechanisms

Ulcerative colitis (UC) is a chronic inflammatory disease of the bowel with unclear etiology. It is characterized by mucous purulent, abdominal pain, and recurrent diarrhea and is a modern refractory disease with an extremely high risk of colorectal cancer (CRC) (20). Currently, immunosuppressants, anti-inflammatory drugs, and biological agents are the main therapeutic approaches for UC. However, it is still difficult to cure because none of the approaches can reverse the colon injury, and a proportion of patients will have recurrent attacks once ceasing the treatments (21). Emerging evidence suggests that traditional Chinese medicine has positive clinical outputs for UC, including reducing recurrent diarrhea, ameliorating intestinal inflammatory responses, and improving the patient’s life quality (22, 23). Given that BBR has a long history in Chinese medicine used as an antibacterial agent to treat dysentery, it is promising to repurpose BBR for UC and other inflammatory-associated diseases. Tang and coworkers have recently demonstrated in a rodent model that oral administration of BBR effectively alleviates animals’ colitis symptoms when combined with another Chinese herb *Atractylodes macrocephala* Koidz (24). The underlying mechanisms involve local- and systemic regulations of the immune system, including the reduced pro-inflammatory cytokines IL-4, IL-6, IL-1β, TNF-α, and myeloperoxidase (MPO), and IgA, IgG levels. Indeed, large-scale genome-wide association studies (GWAS) revealed hundreds of loci associated with UC and implicated genes and core cytokines pathways underlying inflammatory pathology. Such as IFN-γ, IL-17, and IL-13, *etc.*, by which immune cells coordinate their functions and intercellular communications (25). In the dextran sulfate sodium (DSS)-induced colitis mice model, treatment of BBR attenuated all pathologic alterations, especially the suppression of the IFN-γ signaling pathway. BBR treatment consistently down-regulated the IFN-γ targeted genes (*e.g.*, *IRF8*, *IRF1*, *Ifit1* and *Ifit3*) in UC mice. In addition, BBR markedly decreased serum pro-inflammatory cytokines/chemokines IL-17, TNF-α, CXCL1, and CXCL9 levels (26). In contrast, studies demonstrate that BBR can block the excessive pro-inflammatory cytokine production in UC rodents *via* the IL-6/STAT3/NF-κB signaling pathway (27, 28). Following this signaling, BBR exerts antisepsis and antioxidative stress activities by affecting mucosal immunity while improving gut barrier function (27–29). Moreover, the Wnt/β-catenin signaling is pivotal for intestinal epithelial homeostasis and tissue regeneration and is dysregulated during inflammatory responses (30). In line with this, Dong et al. demonstrated that BBR acts as an effective drug for UC treatment in a Wnt/β-catenin signaling-dependent manner (31) where BBR administration maintained intestinal mucosal barrier homeostasis and modulated the colonic T cell response, including the transcription and populations of Th17 and regulatory T cells (Treg).

Notably, in the phase I clinical trial, BBR is shown to lower the Geboes Score (GS, a histological score as a UC indicator) in UC patients from a Chinese cohort (32). Accordingly, it suggests that the GS lowering, inflammation suppression, and tissue-repairing effects of BBR in UC may be mediated *via* the chemosensory Tuft cells-controlled IL-25/C2/13 immune pathway in the colon tissues (33). Meanwhile, Li et al. reported that BBR reduces the colonic infiltration of neutrophils, macrophage and dendritic cells, and innate lymphoid cells (ILCs) and decreases NK cell activation in UC (34). It impedes the colitis from further advancing *via* the JAK-STAT, ERK, and AKT signaling in intestinal stromal cells. Moreover, a protective effect is observed where BBR preserves the colonic mucosal tight junction and modifies the Th17/Treg dynamic equilibrium in DSS-induced colitis mice (35). In addition, the crosstalk of enteric glial-intestinal epithelium-immune cells has been suggested in the BBR regulation of colitis, where Th17 inhibition is a key component (36, 37). With a similar pathway, in another intestinal lesion model induced by cecal ligation and puncture, BBR is demonstrated to exert a protective effect on cecal ligation and puncture (CLP)-induced intestinal injury by reducing the pro-inflammatory response (38). The mechanisms of BBR’s mediation should result from the accumulated proportion of Treg cells and CTLA-4 linked cell-cell contact pathway. Shaping of intestinal macrophage function is a key element of infection resistance and tissue reparation. Therefore, it plays a dominant role in UC pathogenesis and regulation. In this regard, maintaining macrophage polarization homeostasis is critical for UC treatment (22). It is worth mentioning that BBR has been validated to target macrophage polarization and its downstream regulation in health and inflammation; therefore could be a potential therapeutic approach for UC (20, 39, 40).

Microbiology studies in human and animal models have shown that UC stems from skewed immune responses toward one’s commensal microflora or microbiota dysbiosis. In contrast, numerous studies indicate that maintaining gut microbiota homeostasis or providing beneficial microbes/probiotics can substantially improve mucosal barrier function and ameliorate UC (20, 41). Intriguingly, BBR regulates intestinal microbiota, possibly *via* boosting *Blautia* sp., *Lactobacillus* sp., *Bacteroides* sp., *Bifidobacterium* sp., and *Akkermansia* sp. growth while inhibiting the pathogenic bacteria *Enterococci* sp. and *Escherichia coli* in mice with inflammation (42). It is worth noting that BBR has been shown to improve gut tight junction (TJ) protein expression and reduce the Th17/Treg ratio in DSS-induced colitis by promoting intestinal *Bacteroides fragilis* and the associated IL-6 inhibition (35). Again, BBR could modulate intestinal microecology by boosting specific microflora (*e.g.*, *bifidobacteria*), and enriching bacterial fermentation. Therefore, BBR-promoted gut microbiota balance facilitates its protection of intestinal mucosa and barrier integrity in UC (33). Because gut microbiota is vulnerable to high-fat diets, BBR effectively ameliorates the expression of genes involved in short-chain fatty acids synthesis, improves mucosal immunity, and enhances the host inflammatory response against gut lesions induced by the high-fat challenge (43). In contrast, BBR-mediated actions are sensitized to the gut microbiome. For example, BBR weakens the generation of trimethylamine by microbiota to lessen choline-induced atherosclerosis in mice (44). With the enrichment of quote-generating gut microbiome, BBR attenuates ovariectomy-triggered anxiety-like illness. In a human study, BBR exhibits an antidiabetic function in type 2 diabetes by reducing secondary bile acid by repressing *Ruminococcus bromii* growth (45). These studies provide clues of BBR-derived regulations *via* gut microbiome in inflammatory-associated diseases. However, the deep mechanism of BBR on the interaction between gut microbiota and colitis still needs to be explored.

### The anti-tumor activity of BBR and its potential roles in cancer therapy

The antitumor actions of BBR mainly include inducing tumor cell apoptosis, suppressing cancer cell proliferation *via* cell cycle arrest, autophagy, scavenging free radicals, and inhibiting the metastasis of tumor cells without causing overt side effects on normal cells (46, 47). In comparison, a number of pathways of these actions have been studied such as inhibition of the PI3K/AKT/mTOR, Wnt/β-catenin, MAPK/ERK, EGF receptor, Her2/neu, and the VEGF receptor signaling along with induction of Cip1/p21, Rb expression, p53, and Kip1/p27. These are associated with BBR’s anti-inflammation and antioxidant properties (46, 48). It is well-known that chronic inflammation is one of the main factors to cause human cancers (49). And cancer-linked inflammation indicates the seventh hallmark of cancer development and progress (49). In this process, tumor-infiltrating immune cells produce inflammatory mediators involving cytokines, reactive oxygen species (ROS), and free radicals, resulting in a pre-malignant state (50). Subsequently, the released pro-inflammatory cytokines and growth factors stimulate signaling pathways like PI3K/AKT/mTOR, MAPK/ERK, STAT3, and NF-κB. By inhibiting these cascades, medicinal plants or their bioactive extracts, including BBR, can have a preventative effect on tumor onsets (51).

Cancer development is observed when AKT/PI3K/mTOR pathway is activated. At the same time, BBR performs a vital function in tumor management by strongly suppressing the PI3K/AKT/mTOR signaling (46). In a study of gastric cancer, BBR is validated to increase cellular apoptosis, blocks PI3K/AKT/mTOR, and causes the dephosphorylation of the AKT and p38 pathways (52). Inflammation-linked cancer could produce several chemokines and cytokines *via* NF-κB, which directly binds to the specific gene promoters (53). BBR administration efficiently decreases the NF-κB signaling accompanied by pro-inflammatory cytokines IL-1, IL-6, and TNF-α productions (54). It has been noted that BBR drastically suppresses lung cancer cell proliferation *via* NF-κB/COX-2 (55). Furthermore, BBR reduces the activation of the NF-κB pathway *via* enhancing IκBα and inhibits the elevated phosphorylation of c-Fos/Jun in the scratched cancer cells MDA-MB-231 (56). Again, the pro-inflammatory cytokines, interleukins and TNF-α are all suppressed in response to BBR treatment in TNBC cells, this would further inhibit the tumor metastasis (57). Moreover, BBR blunts cancer metastasis of melanoma cells by the reduction of ERK signaling and the activation of the AMPK pathway (58). In agreement, BBR-activated AMPK is a dominant reason to inhibit colorectal carcinogenesis, which could suppress the growth of a colon xenograft tumor when AMPK is activated *via* phosphating AMPK signaling at Thr172 (59). Interestingly, p53 and p38 AMPK are also reported functioning in antitumor processes with BBR treatment (60, 61). In contrast, when BBR inhibits the MAPK/mTOR/p70-S6K pathway, gastric cancer cell growth is markedly suppressed due to cytostatic autophagy (62). It is noteworthy that Wnt/β-catenin signaling activation is strongly associated with CRC initiation (63). BBR presents a strong cytostatic efficiency against human CRC *via* blocking the Wnt/β-catenin pathway to stimulate the caspase‐dependent apoptosis and diminish cancer cell survival (64). This further inhibits the metastasis of CRC because of the cell cycle arrest at G1/S and G2/M phases, DNA damage, and topoisomerase poisoning in colon tumor cells (65).

It suggests that macrophages play complicated roles in cancer depending on cytokines derived from the microenvironment (20). Notably, it has been reported that BBR manipulates the macrophage polarization, reducing the IL-10 and TGF-β pathways in a mouse melanoma model to reinstall their anti-tumor immune responses (66). By increasing the MHC-II and CD40 expression on macrophages, BBR also activates the cytotoxic T lymphocytes (CTL) activity and stimulates the CD4^+^ T-cells derived IFN-γ production (66). Furthermore, BBR performs anti-tumor roles in diffuse large B cell lymphoma (DLBCL) related to rituximab-based immunochemotherapy and CD47-targeted immunotherapy (67). BBA exerts a remarkable synergistic action to enhance the CD47 inhibition resultant-tumor repression by c-myc and promote the phagocytosis of macrophages (67). Finally, BBR could prevent lung cancer by modulating the peptidylarginine deiminase 4 (PADI4)-related macrophage inflammatory responses by up-regulating CD86 and decreasing CD163 and CD206 in the PADI4 overexpressed macrophages (68).

Gut bacteria are tightly linked to cancer oncogenesis and progression, while BBR has exhibited therapeutic potential in bacteria-induced cancer (69). BBR maintains *Fusobacterium nucleatum*-induced intracellular signaling pathways and reduces the secretion of mucosal immune factors, including IL-21, IL-22, IL-31, and CD40 (70). Therefore, BBR facilitates intestinal microbiota homeostasis by increasing *Tenericutes* and *Verrucomicrobia* populations and reduces *F. nucleatum* colonization. BBR modulates the intestinal microbiome by regulating sodium butyrate production and inhibits colon cancer (71). BBR boosts the α and β diversity of microbiota, and the abundance of Bacteroidetes and Proteobacteria, whereas alters the biomarkers and metabolic outputs of the intestinal microbe and decreases the abundance of *Ruminococcus* (71). Given that immunotherapy is a critical part of cancer treatment, BBR has been validated to function on the immune system, showing great potential in cancer immunotherapy (72). For instance, BBR serves as a dopamine D1- and D2-like receptor antagonist to diminish IL-6, IL-1β, IFN-γ, and TNF-α production in the LPS-stimulated lymphocytes (73). It is also addressed that BBR boosts autoimmune neuropathy *via* decreasing IL-1 and TNF-α concentrations together with suppressing CD4^+^ T cell proliferation (74). Again, IFN-γ-induced indoleamine-2,3-dioxygenase 1 (IDO1) expression is reduced when BBR causes the inhibition of STAT1 phosphorylation (75). Moreover, BBR inhibits the PD-1/PD-L1 pathway by inactivating CSN5 deubiquitination in non-small-cell lung carcinoma (NSCLC) and improves anti-cancer T-cell immunity (9). It suggests a rationale for the therapeutic potential of BBR, which can be used as an efficient antagonist of PD-L1 in cancer immunotherapy.

### Conclusions and perspectives

**Figure 1:**
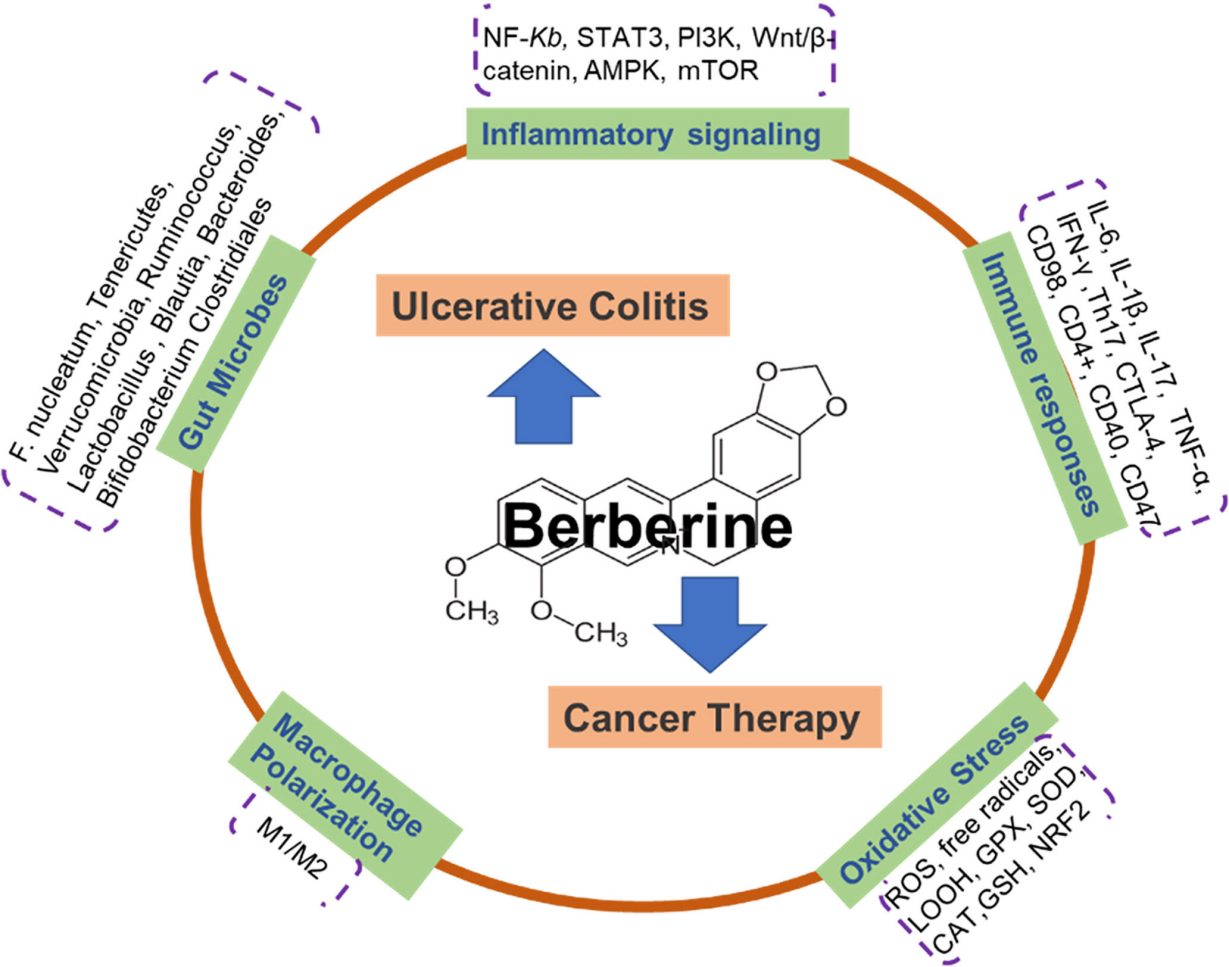
Schematic illustration depicting that berberine actions on ulcerative colitis and cancer therapy *via* multiple mechanisms. Berberine is an isoquinoline alkaloid purified from Chinese herbs and a naturally occurring compound extracted from Coptidis Rhizoma. There are 5 potential functions of BBR on the treatment of ulcerative colitis and cancer, including: inflammatory signaling pathways (NF-κB, STAT3, PI3K, Wnt/β-catenin, AMPK, mTOR); gut microbes (*F. nucleatum*, *Tenericutes*, *Verrucomicrobia*, *Ruminococcus*, *Lactobacillus*, *Blautia*, *Bacteroides*, *Bifidobacterium*, *Clostridiales)*; immune responses (IL-6, IL-1β, IL-17, TNF-α, IFN-γ, Th17, CTLA-4, CD98, CD4+, CD40, CD47); macrophage polarization; Oxidative Stress (ROS, free radicals, LOOH, GPX, SOD, CAT, GSH, NRF2).

In the past several decades, we have witnessed a tremendous advance in exploring the potential mechanisms behind the pathogenesis of ulcerative colitis and cancer therapy. Nevertheless, the therapeutic approaches are still waiting for the findings of more reliably targetable players and available administrators. BBR is a multi-functional herbal medicine. The characteristics of BBR offer it a pivotal candidate for inflammation-associated UC and cancer treatment and attract more attention to study its targets and modes. In this mini-review, we summarized the latest advances in the main actions of BBR on inflammation and immune responses in UC and cancer research. As shown in the schematic diagram (**Figure 1**), inflammatory and immune factors include the signaling pathways of MAPK, NF-κB, Akt, AMPK, and Wnt/β-catenin interleukins, TNF-α, CD4^+^, CD40, and gut microbes, as well as macrophage polarization, are addressed. Although BBR exerts the marked repression of various targets as aforementioned in basic research, the preventive and therapeutic use of BBR against UC and cancer must be explored and validated in clinical studies.

## Author contributions

DC and H-YL: conceptualization. CZ, KL, PH, XP, ZW, and T-JY: writing the original draft. H-YL, and DC: review and editing. All authors contributed to the article and approved the submitted version.
